# Arabidopsis *AtHB7* and *AtHB12* evolved divergently to fine tune processes associated with growth and responses to water stress

**DOI:** 10.1186/1471-2229-14-150

**Published:** 2014-05-31

**Authors:** Delfina A Ré, Matías Capella, Gustavo Bonaventure, Raquel L Chan

**Affiliations:** 1 Instituto de Agrobiotecnología del Litoral, Universidad Nacional del Litoral, CONICET, CC 242 Ciudad Universitaria, 3000 Santa Fe, Argentina; 2 BASF Plant Science, Technologiepark 21C, B-9052, Ghent, Belgium

**Keywords:** AtHB7, AtHB12, Homeodomain-leucine zipper (HD-Zip I), Moderate water stress, Yield, Plant growth

## Abstract

**Background:**

Arabidopsis AtHB7 and AtHB12 transcription factors (TFs) belong to the homeodomain-leucine zipper subfamily I (HD-Zip I) and present 62% amino acid identity. These TFs have been associated with the control of plant development and abiotic stress responses; however, at present it is not completely understood how AtHB7 and AtHB12 regulate these processes.

**Results:**

By using different expression analysis approaches, we found that *AtHB12* is expressed at higher levels during early *Arabidopsis thaliana* development whereas *AtHB7* during later developmental stages. Moreover, by analysing gene expression in single and double Arabidopsis mutants and in transgenic plants ectopically expressing these TFs, we discovered a complex mechanism dependent on the plant developmental stage and in which *AtHB7* and *AtHB12* affect the expression of each other. Phenotypic analysis of transgenic plants revealed that *AtHB12* induces root elongation and leaf development in young plants under standard growth conditions, and seed production in water-stressed plants. In contrast, *AtHB7* promotes leaf development, chlorophyll levels and photosynthesis and reduces stomatal conductance in mature plants. Moreover *AtHB7* delays senescence processes in standard growth conditions.

**Conclusions:**

We demonstrate that AtHB7 and AtHB12 have overlapping yet specific roles in several processes related to development and water stress responses. The analysis of mutant and transgenic plants indicated that the expression of *AtHB7* and *AtHB12* is regulated in a coordinated manner, depending on the plant developmental stage and the environmental conditions. The results suggested that *AtHB7* and *AtHB12* evolved divergently to fine tune processes associated with development and responses to mild water stress.

## Background

Transcription factors (TFs) are proteins able to recognize and bind specific DNA sequences (*cis-*acting elements) present in the regulatory regions of their target genes. These proteins have a modular structure and exhibit at least two types of domains: a DNA binding domain and a protein-protein interaction domain which mediates, directly or indirectly, the activation or repression of transcription [1,2].

TFs play key roles in regulating signal transduction pathways and, in plants, they are main actors in the responses to environmental variations with consequences in growth and differentiation. Some TFs are regulated by one or more abiotic stress factors such as cold, heat, drought and salinity, which suggests pathway cross-talk [3,4].

Around 2000 TFs have been identified in *Arabidopsis thaliana* and 1600 in rice (*Oryza sativa*), which represents 6% and 3% of the total number of predicted genes in these species, respectively [5-7]. However, only a small number of these TFs has been functionally studied so far [4]. TF families are classified according to their binding domain and divided in subfamilies according to additional structural and functional characteristics [5,8].

Within plant TFs, homeodomain-leucine zipper (HD-Zip) proteins constitute a family characterised by the presence of a homeodomain (HD) associated with a leucine zipper (LZ), a combination unique to plants [9-12]. The HD-Zip family has been divided into four subfamilies (I–IV) according to sequence similarity and the intron/exon patterns of the corresponding genes [11,13]. Members of subfamily I interact *in vitro* and *in vivo* with the pseudo-palindromic sequence CAAT(A/T)ATTG [13-16], and have been involved in the adaptive response to abiotic stress [4,11]. Their expression is regulated by drought, salt, abscisic acid (ABA), ethylene, jasmonic acid, freezing and other external conditions and hormones in different tissues and organs [13,16-24].

The HD-Zip domain is highly conserved in subfamily I members from mosses to dicots and monocots but recently our group has reported the existence of uncharacterized conserved motifs outside the HD-Zip identified as putative phosphorylation, sumoylation and transactivation motifs [25]. These motifs, mostly located in the carboxy-terminal regions and to a minor extent in the amino-terminal regions, are, at least in part, responsible for the different functions exerted by these proteins [25]. The importance of the carboxy-terminal motifs in these TFs function has been deeply analysed, indicating that the mutation of individual amino acids in these motifs significantly affect their ability to activate and to interact with proteins of the basal transcriptional machinery [26]. The HD-Zip subfamily I has 17 members in Arabidopsis that have been classified into six groups according to their phylogenetic relationships and gene structure, including introns number and location [13]. More recently, a phylogenetic reconstruction with 178 HD-Zip I proteins from different species was performed. In this new phylogenetic analysis, that considers the conserved motifs in the carboxy-terminal regions, Arabidopsis members are classified in six groups, named I to VI [25].

In this new classification, AtHB7 (*Arabidopsis thaliana* Homeobox 7) and AtHB12 (*Arabidopsis thaliana* Homeobox 12)*,* which present 62% amino acid identity, have been defined as paralogues belonging to group I. Interestingly, a new homology search using their sequences as query has revealed that for most species, AtHB12 and AtHB7 indistinctly match to only one HD-Zip I. *Capsella rubella*, a *Brassicaceae* species, was the exception presenting two HD-Zip I (CARUB10017952 and CARUB10023896) matching with AtHB12 and AtHB7, respectively [25]. As examples, MtHB1 (*Medicago truncatula* Homeobox 1) [16] and NaHD20 (*Nicotiana attenuata* Homeodomain 20) [27] are unique for this clade in these species. Hence, with the current knowledge, it can be suggested that AtHB12 and AtHB7 as well as AtHB5 and AtHB6, respectively, diverged from a common ancestor in *Brassicaceae.* Among Arabidopsis HD-Zip I transcription factors, AtHB6 and AtHB5 were well characterised; AtHB6 has been described as a positive regulator of ABA responsive genes being targeted by CRL3 (Cullin-RING E3 ubiquitin Ligases 3) [24] while AtHB5 is a negative regulator of auxin-related genes [28]. The expression of *AtHB7* and *AtHB12* has been detected by Northern blots in meristems, root tips and flowers and a strong up-regulation has been observed after osmotic or drought stresses and when young 14-day-old plants were treated with ABA or NaCl [18,29]. Olsson *et al*. [18] have postulated that *AtHB7* and *AtHB12* are negative developmental regulators in response to drought. Moreover, based on the characterization of mutant and overexpressor plants on Ler (Landsberg) and WS (Wassilewskija) backgrounds, *AtHB12* has been assigned a role as regulator of shoot growth in standard growth conditions [30]. On the other hand, the ectopic expression of *AtHB7* in tomato confers drought tolerance to this species [31]. In another report, loss-of-function *athb7* and *athb12* mutants have revealed that both genes activate clade A protein phosphatases 2C (PP2C) genes and reppress *PYL5* and *PYL8 (Pyrabactin Resistance 1-like* 5 and 8*), ABI1 (ABA Insensitive 1)*, *ABI2 (ABA Insensitive 2)*, *HAB1 (Hypersensitive to ABA 1)*, *HAB2 (Hypersensitive to ABA 2)*, and *PP2AC* or *AHG3 (Protein Phosphatase 2CA)*, thus acting as negative regulators of ABA signaling [32]. It is noteworthy that the binding of some of these targets is ABA-dependent for AtHB12 but not for AtHB7 [32].

Summarizing, even though several studies have significantly contributed to the understanding of the regulation of *AtHB7* and *AtHB12* expression in Arabidopsis, most of the studies were performed with different Arabidopsis genotypic backgrounds and taking only one of both genes as subject [18,29,30,32-36]. Though, many aspects of their function in plant development and in response to water availability remain unknown. Even more important, it is unclear what the biological significance of the recent duplication of these two genes is, how specifically/redundantly they act and how they affect plant homeostasis. In this study, we aimed at bringing light to some of these aspects.

## Results

### The expression of the duplicated genes, *AtHB7* and *AtHB12,* is coordinated during development

**Figure 1:**
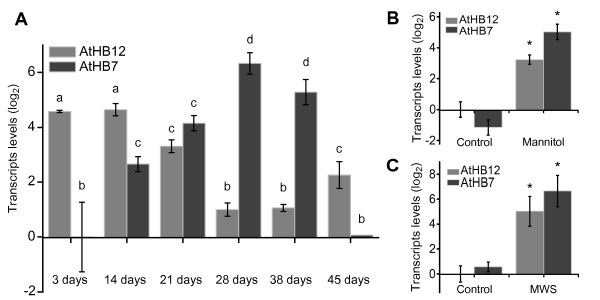
***AtHB12 *and *AtHB7 *expression levels fluctuate during development and in response to abiotic stress. (A)** Total RNA was isolated from 3- and 14-day-old WT plants and from leaves of 21, 28, 38 and 45-day-old plants and analysed by qRT-PCR for *AtHB7* and *AtHB12* transcript levels with specific oligonucleotides (Additional file 4). **(B)** Total RNA was isolated from 14-day-old plants treated with 300 mM mannitol and analysed as in A. **(C)** RNA was isolated from leaves of 38-day-old plants subjected to a moderate water stress (MWS) starting at day 21 after germination, during 17 days. Transcript levels values were normalised with AtHB7 transcripts at day 3 in A or at time 0 in B and C, applying the ∆∆Ct method. Error bars represent SE calculated from three independent biological replicates. Actin transcripts (*ACTIN2* and *ACTIN8*) were used as a reference. “*” , “a”, “b”, “c” and “d” denote statistical differences obtained with-ANOVA-Tukey’s P < 0.05.

Aiming at knowing how these two genes are expressed during the plant life cycle, transcript levels of both *AtHB7* and *AtHB12* were first quantified in wild type (WT) Arabidopsis Col-0 (Columbia) ecotype at different growth stages. RNA was purified from 3-day-old seedlings and rosette leaves of 14, 21, 28, 38 and 45-day-old plants and transcript levels were quantified by qRT-PCR. In seedlings, *AtHB12* transcripts levels were 16 times higher than in leaves of 28- to 45-day-old plants while *AtHB7* transcripts were 30 times lower in seedlings than in leaves of 28-day-old and slowly decreased after this stage (Figure 1A). As shown in Figure 1A, the expression patterns of *AtHB12* and *AtHB7* were opposite; when one of them was highly expressed, the other was repressed. The expression levels of *AtHB12* and *AtHB7* were also analysed in response to osmotic stress induced by mannitol on 14-day-old plants. After this treatment, *AtHB12* and *AtHB7* transcript levels were induced 8- and 30-fold, respectively (Figure 1B). A moderate water stress (MWS) treatment was also applied to soil-grown 45-day-old plants and consistent with the mannitol treatment, the transcript levels of both TFs were also induced (ca. 30-fold; Figure 1C).

To substantiate the gene expression results observed by quantification of mRNA levels, transgenic plants carrying C-terminal protein fusions between *AtHB7* or *AtHB12* and *Green Fluorescent Protein* (*GFP*) - β*-GLUCURONIDASE* (*GUS*) were generated. Aiming at reflecting as much as possible the real biological scenario, genomic fragments encompassing *AtHB7* and *AtHB12* promoter regions and coding sequences were cloned upstream of the reporter genes.

**Figure 2:**
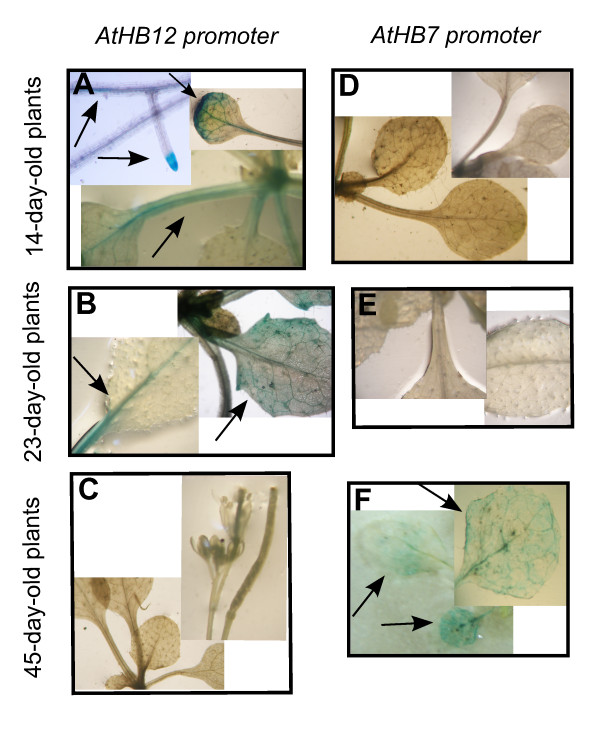
***GUS *expression directed by *AtHB7 *or *AtHB12 *promoters depends on the stage of development.** Histochemical detection of GUS enzymatic activity in *pAtHB7::GUS* and p*AtHB12*::GUS plants of 14-, 23- and 45-day-old as indicated. **(A)**, **(B)**, **(C)**: *AtHB12* promoter and **(D)**, **(E)**, **(F)**: *AtHB7* promoter. View of root tips, cotyledons, petioles and nervations **(A)**; petioles and nervations **(B)**; leaves, flowers and siliques **(C)**; leaves and cotyledons **(D and E)**; senescent leaves **(F)**.

These transgenic plants were named p*AtHB12:AtHB12::GFP::GUS* and p*AtHB7:AtHB7::GFP::GUS*, respectively, and were analysed histochemically. The analysis was performed on 14-, 23- and 45-day-old plants, all grown in standard conditions (see Methods). As shown in Figure 2, *AtHB12* promoter activity was clearly detected in roots and leaves of 14 and 23-day-old plants (A and B) but not in 45-day-old plants (Figure 2C). In contrast, *AtHB7* promoter activity was only detectable in senescent leaves of 45-day-old plants (F) but not at earlier stages (D and E). Both, RNA expression and histochemical assays indicated that *AtHB12* transcripts were particularly abundant during early developmental stages while *AtHB7* during later stages. Such spatio-temporal differences in the expression of these TFs suggested that *AtHB12* and *AtHB7* have specific rather than redundant functions in plant growth and development.

### AtHB7 and AtHB12 affect the expression of each other during development in standard growth conditions

Considering the almost opposite expression patterns of *AtHB7* and *AtHB12* during plant development in standard growth conditions (Figures 1A and 2), we investigated whether the expression of these TFs could influence each other. First, transient transformation of *Nicotiana benthamiana* leaves was conducted with the purpose of analysing whether AtHB7 and AtHB12 affected the activity of their paralogs’ promoter. Transient co-transformation of leaves by syringe-infiltration [37] was performed with *Agrobacteria* carrying the constructs p*AtHB7:AtHB7::GFP::GUS* or p*AtHB12:AtHB12::GFP::GUS* and a construct in which each TF cDNA was under the control of the *35S CaMV* (*Cauliflower Mosaic Virus*) promoter (*35S::AtHB12* and *35S::AtHB7*). Similar to Arabidopsis transgenic plants, genomic fragments encompassing *AtHB7* and *AtHB12* promoter regions and coding sequences were cloned upstream of the reporter genes. As negative and positive controls, *pBI101.3* (*non promoter::GUS*) and *pBI121* (*35S::GUS*) were used, respectively. Two days after leaf infiltration, *GUS* transcript levels were quantified by qRT-PCR.

**Figure 3:**
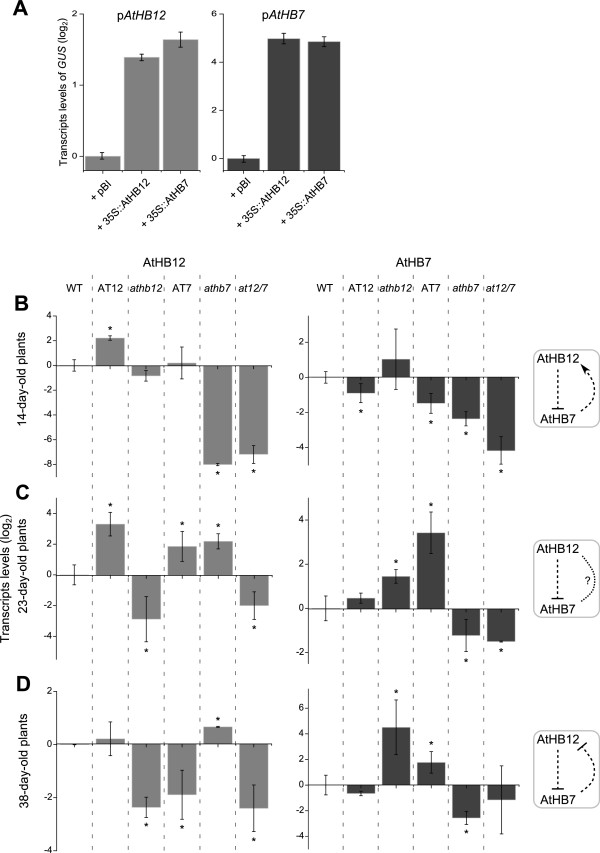
***AtHB12 *and *AtHB7 *regulate each other along development in standard growth conditions.** Total RNA was isolated from mutant and WT plants (indicated in the top). *AtHB12* and *AtHB7* transcript levels were analysed at three different developmental stages (14-, 23- and 38-day old plants); a scheme of the proposed effect of each TF on the other is shown on the right (positive → or negative --/ effect). **(A)** Transcript levels of *GUS* after transient co-transformation of *N. benthamiana* leaves with *A. tumefaciens* carrying p*AtHB12::GFP::GUS* or p*AtHB7::GFP::GUS* and the constructs indicated in the x axis, quantified by qRT-PCR. Values were normalised with respect to that measured in control samples (p*AtHB12::GFP::GUS or* p*AtHB7::GFP::GUS + pBI 101.3*) by ∆∆Ct method. **(B)** Transcripts levels in 14-day-old plants. **(C)** Transcripts levels in 23-day-old leaves. **(D)** Transcripts levels in 38-day-old leaves. Transcript levels were quantified by qRT-PCR and the values normalised with respect to that measured in WT plants applying the ∆∆Ct method. Error bars represent SE calculated from three independent biological replicates. Actin transcripts (*ACTIN2* and *ACTIN8*) were used as a reference. “*” denotes statistical differences obtained with one-way-ANOVA-Tukey’s P < 0.05.

Leaves co-transformed with *pAtHB12:AtHB12::GFP::GUS* plus *35S::AtHB7* or plus *35S::AtHB12* expressed *GUS* at approximately 2-fold higher levels than leaves co-transformed with p*AtHB12:AtHB12::GFP::GUS* plus *pBI101.3* (Figure 3A). When leaves were co-transformed with p*AtHB7:AtHB7::GFP::GUS* plus *35S::AtHB12* or plus *35S::AtHB7*, *GUS* expression was approximately 6-fold higher than in control leaves co-transformed with p*AtHB7:AtHB7::GFP::GUS* plus *pBI101.3* (Figure 3A).

These results indicated that in the tobacco heterologous transient system, the ectopic expression of either AtHB12 or AtHB7 positively affects the activity of their own promoter and of their paralogs’ promoter. However, even though both genes exhibit in their regulatory regions some elements partially matching the pseudopalindromic sequence CAAT(A/T)ATTG (bound *in vitro* by all the HD-Zip I tested so far [14,15]), a transient transformation assay in a heterologous system provides only partial evidence of a potential direct interaction between the tested TFs and their promoters. Thus, to further investigate the putative effect of AtHB12 and AtHB7 on the expression of each other, single mutants (*athb12* and *athb7*), a double knock-down mutant (*at12/7*) and overexpressors of each of these genes (AT12 and AT7) were obtained and characterised.

Transcript levels of *AtHB7* and *AtHB12* were quantified in all the genotypes and control plants at three different developmental stages. In 14-day-old plants, *AtHB12* presented almost the same expression levels in AT7 as in WT plants but expression was almost undetectable in *athb7* plants. Notably, *AtHB7* transcript levels in AT7 plants were lower than in WT plants during this developmental stage, which is worth noting since in AT7 plants, *AtHB7* expression is driven by the *35S CaMV* promoter. Thus, based on this observation, it is tempting to speculate that *AtHB7* transcripts are degraded in the overexpressor lines by the triggering of post-transcriptional gene silencing mechanisms [38]. Moreover, at this developmental stage, AT12 plants exhibited 2-fold lower *AtHB7* mRNA levels than WT (Figure 3B). Altogether, these observations suggested that AtHB12 may repress *AtHB7* expression and, on the other hand, that AtHB7 induces *AtHB12* expression at the transcriptional level in the vegetative stage (Figure 3B).

Twenty three days after germination, the plants already transitioned to the reproductive phase under the growth conditions used for this study. At this stage, *AtHB12* transcripts were 4-fold higher, both in AT7 and *athb7* plants compared with WT plants. *AtHB7* exhibited similar transcript levels in AT12 and WT plants and higher levels in *athb12* and AT7 plants compared to WT plants (Figure 3C). These observations suggested that AtHB12 somehow down-regulated *AtHB7* expression while AtHB7 did not affect *AtHB12* expression at this developmental stage (Figure 3C).

The scenario changed in 38-day-old plants; *AtHB12* transcript levels were 4-fold lower in AT7 and 2-fold higher in *athb7* than in WT plants (Figure 3D). *AtHB7* transcript levels were 4-fold and 16-fold higher in AT7 and *athb12,* respectively, than in WT plants and 1.3-fold lower in AT12 than in WT plants. At 23- and 38-day-old, AT7 plants exhibited high *AtHB7* transcript levels as it is expected when plants are transformed with constitutive promoters like the *35S CaMV* (Figure 3D).

To summarize, the results presented so far could be interpreted by the scheme shown in the right panel of Figure 3. This scheme illustrates that at early developmental stages, AtHB7 positively regulates *AtHB12*, and that AtHB12 negatively regulates *AtHB7.* In mature plants, the effect observed is a double negative feedback loop between *AtHB7* and *AtHB12*. These results, together with those obtained by *N. benthamiana* transient co-transformation, suggest a complex regulation of *AtHB7* and *AtHB12* expression, changing during development and requiring the participation of additional factors. However, it is necessary to understand if this regulation or coordination between AtHB7 and AtHB12 has a functional purpose.

### Changes in *AtHB7* and *AtHB12* expression affect seedling root growth, bolting time and leaf growth

The differential pattern of *AtHB7* and *AtHB12* expression observed in Arabidopsis during development led us to investigate the physiological processes controlled by these two TFs. For this purpose, a deep phenotypic characterization of mutant and overexpressor plants in standard growth conditions was conducted.

**Figure 4:**
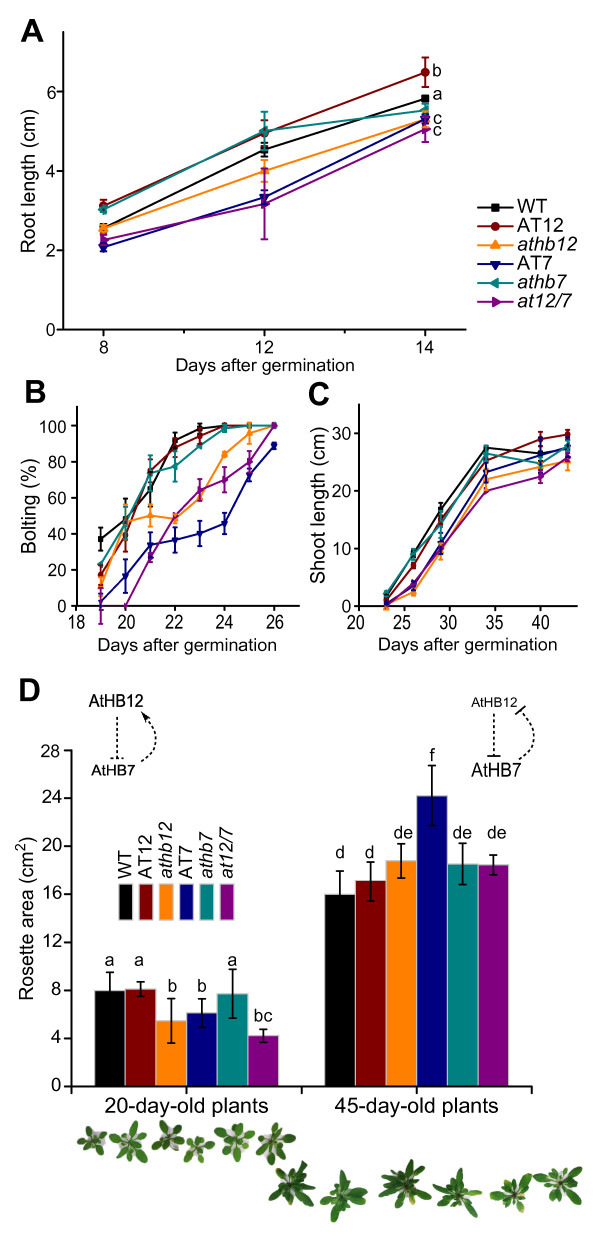
***AtHB12 *and *AtHB7 *contribute to control roots elongation, bolting time, shoots length and leaves development. (A)**. Roots length (cm) of 8- to 14-day-old plants grown in standard conditions. **(B)** Percentage of bolted WT and mutant plants since 19 to 26 days after germination. **(C)** Shoot length (cm) analysed during plant development (between days 23 to 45). **(D)** Total rosette area of 20- and 45-day-old plants. Illustrative pictures of rosettes of each genotype are shown at the bottom. Error bars represent SE (A: n = 10; B: n = 3 independent assays with 8 plants per genotype each assay; C and D: n = 8); “*”, “a”, “b”, “c”, “d”, “e” and “f” denote statistical differences obtained with one-way-ANOVA-Tukey’s P < 0.05.

Plants were grown on MS-Agar plates and roots of 8 to 14 day-old seedlings of AT12, AT7, *athb12*, *athb7* and *at12/7* genotypes were analysed. AT12 seedlings exhibited 15–20% longer roots while AT7 and *at12/7* had 20% shorter primary roots than WT plants. Mutant *athb12* and *athb7* genotypes did not showed statistically significant differences (Figure 4A).

Developmental stages in Arabidopsis can be generally divided in vegetative (before bolting) and reproductive (after bolting) [39]. Bolting occurred at day 22 for 80% of WT, AT12 and *athb7* plants while this event occurred at day 25 (3 days later) for 80% of AT7, *athb12* and *at12/7* plants (Figure 4B). AT7, *athb12* and *at12/7* plants showed a delay in shoot elongation at the beginning of the life cycle but this difference disappeared at later stages and the height of the stems were similar in all genotypes (Figure 4C). The rosette area of 20-day-old plants from genotypes *athb12,* AT7 and *at12/7* was 25% smaller than WT. In 45-day-old plants, the AT7 genotype exhibited 50% larger rosettes than WT while AT12 rosettes were similar to those of WT (Figure 4D). Altogether it can be concluded that in early stages, AtHB12 is necessary for proper growth of rosette leaves but this role is undertaken by AtHB7 at later stages. These data suggested similar roles but at different developmental stages, for these HD-Zip I TFs.

### Differences in *AtHB7* and *AtHB12* expression affect chlorophyll content, photosynthesis rate and senescence

**Figure 5:**
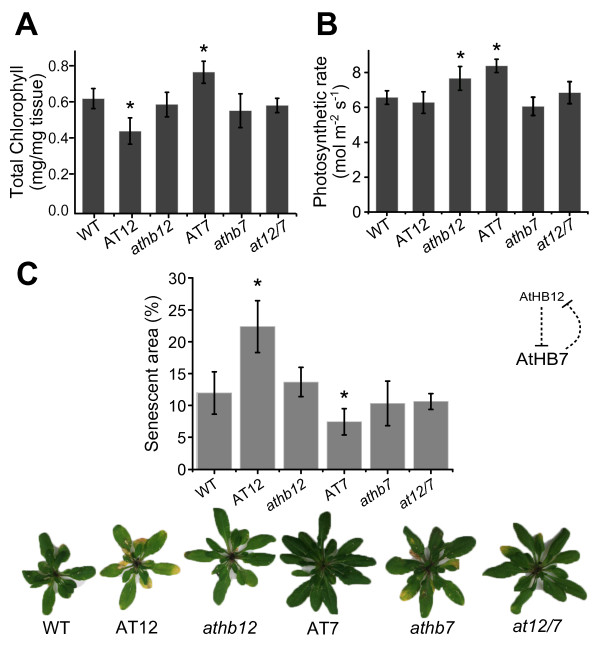
**Chlorophyll content, photosynthesis rate and senescence time are regulated by *AtHB7 *and *AtHB12 *in mature plants. (A)** Total chlorophyll content quantified in 45-day-old plants leaves. Extracts were prepared from green rosette leaves of plants grown under standard conditions during 45-days. **(B)** Photosynthetic rate quantified with IRGA in leaves of 45-day-old plants. **(C)** Senescence degree as the percentage of yellow area in the rosette quantified after scanning with ImageJ. Illustrative photographs of 48-day-old rosettes of each genotype. Error bars represent SE (n = 5); “*” denotes statistical differences obtained with one-way-ANOVA-Tukey’s P < 0.05.

Considering the differences in leaf-area observed between mutant and overexpressor plants (Figure 4D), we investigated whether these differences were also reflected in photosynthesis rate and/or chlorophyll content. Chlorophyll content was similar in WT, mutant and overexpressor 20-day-old plants (data not shown), but 45- day-old AT7 and AT12 plants (among all the genotypes) exhibited significant differences. AT7 showed a 15% chlorophyll increase per mg of leaf tissue while AT12 a 15% decrease, both compared to WT (Figure 5A). Using an Infrared Gas Analyzer (IRGA), photosynthetic rates were analysed. Forty five-day-old AT7 and *athb12* plants exhibited respectively 25% and 15% higher photosynthetic rates (measured as the exchanged CO_2_ per unit of leaf area [mol m^-2^ s^-1^] than controls and other mutant and overexpressors (Figure 5B). In addition to the differential photosynthesis rates and chlorophyll content of AT7 plants, senescence was delayed in these plants. Illustrative pictures are shown in Figure 5C. Forty seven-day-old AT12 plants were the most senescent with 23% yellow area relative to the entire leaf area while AT7 plants were the less senescent presenting only a 6% yellow area (Figure 5C). The other genotypes, *athb7*, *athb12* and *at12/7* exhibited around a 10% senescent area at this developmental stage (Figure 5C). These results suggested that AtHB7 delays senescence while AtHB12 induces it.

### Differences in *AtHB12* and *AtHB7* expression affect water uptake, water loss and seed setting during moderate water stress conditions

Knowing that *AtHB12* and *AtHB7* are up-regulated by water and osmotic stress (this work, [13,18], phenotypes related to dehydration responses were analysed. In this sense, stomata number and dynamics, water uptake, water loss, and production of seeds were evaluated in *AtHB7* and *AtHB12* mutant and overexpressor lines.

**Figure 6:**
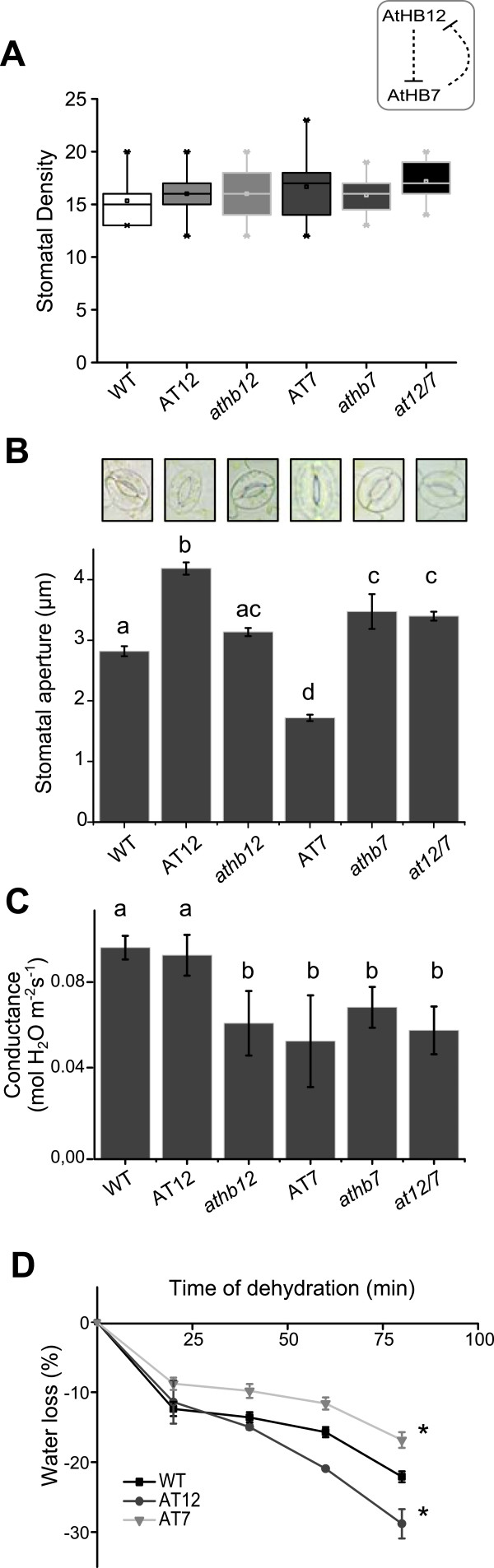
***AtHB7 *induces stomata closure. (A)**. Stomatal density was determined by counting pores per area under microscopy in 38-day-old leaves. **(B)** Stomata’s aperture (μm) was evaluated in three 38-day-old leaves from different individuals per genotype. **(C)** Stomata’s conductance determined by IRGA and expressed as mol H_2_O m^-2^ s^-1^. **(D)** Weight loss in detached 38-day-old leaves evaluated every 20 min by weighting and illustrated as the% of the initial weight. Error bars represent SE (A: n = 5 pictures per genotype; B: n = 15 stomatas from three different leaves per genotype; C: n = 4 leaves per genotype); “*”, “a”, “b”, “c” and “d” denote statistical differences obtained with one-way-ANOVA-Tukey’s P < 0.05.

Stomatal density, quantified as the number of stomata per area unit and stomatal pore aperture were evaluated in leaves of 38-40-day-old plants grown in standard growth conditions (see Methods section). As shown in Figure 6A, the stomata number was similar in mutant and overexpressor lines. Regarding stomata aperture, AT7 plants had on average 30% smaller pores than WT while *at12/7*, AT12 and *athb7* had on average 15–20% bigger pores than WT (Figure 6B).

Water conductance in leaves was quantified by IRGA. AT7, *athb12*, *athb7* and *at12/7* showed lower levels of conductance than WT and AT12 plants (Figure 6C). To evaluate the dynamics of the stomata in response to dehydration conditions, a water-loss assay was performed. Leaves were detached from the plant, placed on tissue paper and weighted every ten minutes to evaluate water loss by transpiration. AT12 leaves exhibited a more pronounced water-loss curve while water loss in AT7 leaves was less pronounced (Figure 6D); *athb12*, *athb7* and *at12/7* plants showed no differences compared to WT (Additional file 1). The results suggested that *AtHB7* induced stomata closure while *AtHB12* induced stomata opening.

### Differences in *AtHB12* and *AtHB7* expression affect seed production under moderate water stress or standard conditions

**Figure 7:**
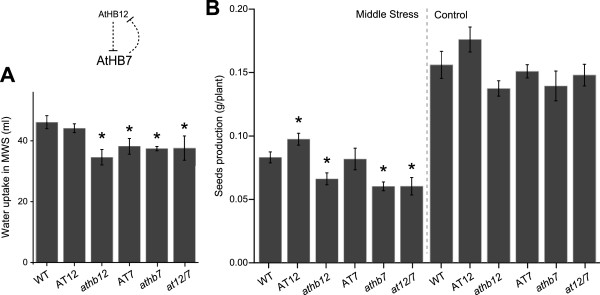
***AtHB12 *and *AtHB7 *are involved in determining water conductance and uptake, and seeds production.** Plants were grown (1 per pot) and MWS treatment was started at day 20. **(A)** Water (ml) added to maintain the same weight in all pots, considered as water uptake during the stress treatment. **(B)** Seeds production in plants grown under MWS conditions (left) or standard conditions (right), as g / plant. Mean is shown and error bars represent SE (A: n = 5; B and C: n = 10); “*” denotes statistical differences obtained with one-way-ANOVA-Tukey’s P < 0.05.

To evaluate water uptake under stress conditions, soil-grown plants were exposed to a moderate water stress (MWS; see Methods section) by irrigating with the minimal volume necessary to maintain pots weight equal during the treatment. Water was applied every 48–72 hours and the needed volume for each genotype added and documented. AT7, *athb12, athb7* and *at12/7* plants needed 20% less water to maintain equal pot weight during the complete MWS treatment compared with WT and AT12 genotypes (Figure 7A). At the end of the life cycle, concomitant with the stress treatment, all produced seeds were harvested and weighted (total seed weight = yield). This quantification showed that *athb12*, *athb7* and *at12/7* yielded 15–20% less than WT and AT7, while AT12 plants yielded 20% more than WT (Figure 7B, left panel). In standard conditions we did not observe statistically significant differences between genotypes in seed production (Figure 7B, right panel).

These results suggested that AtHB12 and AtHB7 have particular functions in Arabidopsis performance during water limiting conditions. Both may coordinate the regulation of each other expression depending on the stage of development and the availability of water.

## Discussion

### Are the paralogs AtHB7 and AtHB12 playing different roles?

**Figure 8:**
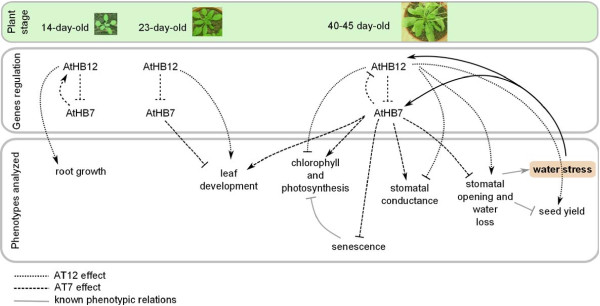
**Schematic representation of the putative roles exerted by *AtHB7 *and *AtHB12 *genes in different developmental stages.** Upper panel: illustrative photographs of plants at the stages they were evaluated. Middle panel: proposed model of regulation of the expression of these genes at three developmental stages (14-, 23- and 40- to 45- day old plants). Lower panel: associations between genes regulation and observed phenotypes.

The results presented in this study support the hypothesis that *AtHB12* and *AtHB7* diverged in Arabidopsis in order to play related yet different functions during development and water stress-related responses. Importantly, these functions are tightly coordinated; these two TFs affect the levels of each other’s expression during development but not in water stress-related responses where both are synchronously induced and play specific roles (Figure 8). The coordinated regulation of the expression of these TFs may require the participation of additional unknown factors.

The information available in databases indicates that AtHB7 and AtHB12 are paralogs that diverged from a common ancestor in *Brassicaceae*[13].These HD-Zip I TFs have been resolved in the group IC of HD-Zip I exhibiting similar, although not identical, motifs outside the conserved HD-Zip domain [25]. The ability of these TFs to activate in plants and yeast systems strongly depends on those differential motifs [26]. Only a single copy gene has been resolved in the same clade in most species analysed so far, with a few exceptions. Within these exceptions are Vv-XP22629 and Vv-CAN7896 from *Vitis vinifera* and Pt-HB7 and Pt-731421 from *Populous trichocarpa*, having two almost identical proteins in this clade [25]. The only characterised exceptions are the paralogous HD-Zip I encoding genes *Vrs1* and *HvHox2* (*Hordeum vulgare Homeobox 1 and 2)* constituting an example of neo-functionalization [40]. These barley HD-Zip I TFs have different expression patterns and play different functions in spikelet development [40]. Like AtHB7 and AtHB12, Vrs1 and HvHox2 differ in their carboxy-termini outside the conserved HD-Zip domain; HvHox2 exhibits 14 additional amino acids compared to VRS1 [40]. The authors suggested that this additional motif could interact with certain classes of co-activators in order to exert their biological function [23]. AtHB7 and AtHB12 exhibit in their carboxy-termini a conserved motif of unknown function and AtHB12 has also a canonical AHA motif [41]. AtHB7 has a divergent transactivation motif and 20 amino acids between the LZ and the conserved motif of unknown function [27]. Additionally, AtHB7 has three putative phosphorylation motifs while AtHB12 has only two [25]. A representation of these structural features is shown in Additional file 2. Even though AtHB7 and AtHB12 present these differences at the amino acid level, differential functions for these two genes have not been assigned yet. AtHB12 binds to some specific targets (*AHG3*, *PYL5* and *PYL8*) only in the presence of ABA while AtHB7 binds the same targets, independently of ABA, indicating functional differences between these TFs [32]. Yeast two-hybrid and pull down assays have shown that AtHB7 interacts with TBP (TATA Binding Protein) and TFIIB (Transcription Factor IIB) from the basal transcriptional machinery while AtHB12 only interacts with TFIIB [26]. The induction of the expression by ABA exhibits a different kinetics for *AtHB12* and *AtHB7*, at least in the Col-0 background [29,33,34]. These previous observations and the differences in amino acid sequence in the C-terminal regions of AtHB7 and AtHB12 together with the results presented in this study, tempted us to speculate that additional factors (e.g., co-repressors) interacting with these TFs via the C-terminal regions and probably operating in a developmental stage-, tissue- and stress-related manner, might participate in the proposed coordinated regulation of *AtHB7* and *AtHB12* expression (Figure 8).

### AtHB12 and AtHB7 expression levels are finely coordinated, contributing to plant development

Here it is shown that the expression of *AtHB7* and *AtHB12* exhibited almost opposite patterns along the Arabidopsis life cycle when plants were grown in standard (i.e. growth chamber) conditions. In early developmental stages (14- to 21-day-old plants), *AtHB12* mRNA was abundant while *AtHB7* mRNA was lower (Figure 1A). In contrast, in advanced developmental stages (21- to 38-day-old plants) the opposite was observed (Figure 1A). The analysis of transgenic plants carrying either *AtHB7* or *AtHB12* promoters directing the expression of the *GUS* reporter confirmed these results (Figure 2). The expression patterns of *AtHB7* and *AtHB12* in *athb7* and *athb12* mutants and AT7 and AT12 ectopic expressors were unexpected and indicated a complex effect of each one on the expression of the other. In this regard, *AtHB7* was expressed only in 23- and 38-day-old AT7 plants, but not in 14-day-old plants. This can be explained by a repression exerted by AtHB12, highly expressed at this developmental stage. Accordingly, *AtHB7* transcripts were clearly detected also in 23- and 38-day-old *athb12* plants (Figure 3B, C and D). This effect of AtHB12 on *AtHB7* expression probably occurs via an indirect mechanism. The results obtained by transient co-transformation pointed out this conclusion (indirect mechanism) because, even when both promoters’ activities were affected by the presence of both TFs, the observed expression changes were opposite to those observed in the mutants.

### Role of AtHB7 and AtHB12 in plant development

The transcriptional coordinated regulation of *AtHB12* and *AtHB7* affects the development of leaves. In AT7 and *athb12* 20-day-old plants, rosette leaf area was reduced compared with WT, while in 45-day-old AT7 plants rosette leaf area was larger than WT’s. Root growth was accelerated in AT12 young plants while AT7 plants showed the opposite roots phenotype (Figure 4). Transcript levels reported here were evaluated in aerial tissues; when roots RNAs were tested, expected overexpression levels were observed (Additional file 3). In *Medicago truncatula*, root development is also controlled by the HD-Zip I TF MtHB1. MtHB1 exhibits a 54% similarity with both AtHB12 and AtHB7. However, considering *mthb1* and *35S::MtHB1* roots architecture [16], it seems that AtHB12 is the orthologue of MtHB1 and that AtHB7 is playing a different role, at least in roots (Figure 4A). This comparison supports that the divergence of these HD-Zip I TFs in Arabidopsis led to a sub-functionalization.

It could be suggested that AtHB7 plays a role in mature 45-day-old leaves since plants overexpressing this TF had more chlorophyll per leaf fresh biomass, increased photosynthesis per leaf unit area, and delayed senescence (Figure 5). On the other hand, *AtHB12* overexpression caused the opposite effect for these parameters at this developmental stage (45-day-old plants). Other authors have reported that the ectopic expression of *AtHB7* induces chlorophyll production in tomato plants [31]. Altogether, these data led us to conclude that AtHB12 has a role promoting root growth and leaf development at the beginning of the life cycle until the plants are approximately 25-day-old while AtHB7 exhibits a major role promoting leaf development, photosynthesis rate and delaying senescence at more advanced developmental stages. The double knock-down mutant *at12/7* did not display a very pronounced phenotype compared to single mutant plants, both in standard and water stress-related conditions. These small differences between the double and the single mutants could most likely be explained by the fact that the *at12/7* double silenced plants had developmental-stage-dependent reduced but not null expression of both genes (Figure 3). In this regard, double silenced *at12/7* and *athb12* 20-day-old mutant plants, exhibited similar phenotypes, slight smaller rosettes and shorter roots compared with WT plants (Figure 4A and D). Accordingly, shorter roots have also been observed in a double mutant *athb12/athb7* by Valdés *et al*. [32].

Olsson *et al*. [18] reported that both TFs affected shoot elongation, leaf morphology and also root growth when 10-day-old plants have been treated with exogenous ABA. *35S::AtHB12* plants had a diminished root growth in ABA and shorter shoots, on WS Arabidopsis ecotype [18]. However, the construct used by these authors to generate transgenic *35S::AtHB12* plants contained an incomplete version of *AtHB12* encoding a truncated protein lacking the carboxy-terminal region [18] (Methods section). In 2004, the importance of the carboxy-termini in HD-Zip I TFs [25] was unknown, the use of that construct instead of a complete one, used in the present work, could explain the discrepant results observed regarding shoot growth. As it was reported [25], carboxy-termini regions in HD-Zip I TFs exhibit conserved motifs playing important roles in these proteins functions. In some cases, the importance of such motifs was experimentally demonstrated [26,40,42] and so, the lack of one or more of them can significantly change the TF activity.

Son *et al*. [30] described an *athb12* mutant exhibiting longer stems compared with WT while in this study stems in this mutant did not differ from WT. These discrepancies could be explained by the genotype used to carry out the experiments (WS vs Col-0). In summary, previous studies suggested similar roles for *AtHB7* and *AtHB12* in inflorescence stems elongation and leaves as well as in stress responses [18,30]. However, those studies characterised separately either *AtHB7* or *AtHB12* in different Arabidopsis genotypic backgrounds, what makes difficult the underpinning of the function of these TFs.

### Role of AtHB7 and AtHB12 in water stress conditions

*AtHB7* and *AtHB12* were described as water stress responsive genes and roles as negative developmental regulators in response to ABA were assigned [18]. Previously reported Northern blot analyses of 10-day-old seedlings have indicated that *AtHB7* and *AtHB12* are induced by ABA and water availability [35]. Later, the same research group showed that these genes promoters are induced by ABA and drought in 30-day-old plants [18]. When osmotic or water stress were applied, both *AtHB7* and *AtHB12* transcripts levels were strongly induced, indicating that activation by these stress stimuli overcomes the feedback loops observed between these two TFs during plant growth and development (Figure 1B and C).

In this work, several parameters related to drought stress were analysed in mature plants. The assays indicated that when plants were water stressed, water loss was induced by *AtHB12* and repressed by *AtHB7* overexpression; water uptake was also affected by the knock-down of these genes and this fact influenced seeds yield (Figures 6D and 7). The apparent discordant observation regarding similar water uptake behaviours in all mutant genotypes and in AT7 plants could be explained if both, AtHB7 and AtHB12, are necessary to optimize this trait. Hence, the absence of any one of these genes affects the expression of the other and provokes a reduction in water consumption.

Water loss and water uptake results were consistent (Figures 6 and 7). AT7 plants consumed less water than WT and lost less water by leaf transpiration (Figures 6D and 7A). On the other hand, *athb7* and *at12/7* plants, compared with WT, took less water during MWS (Figure 7A) but lost the same amount of water during dehydration (Additional file 1). This may be explained by the fact that, among these two genes, AtHB7 seems to be the responsible for stomata aperture and in *athb7* and *at12/7* plants it is absent. These results support very specific roles of these TFs in plants subjected to water stress.

It is noteworthy that while AT12 plants exhibited less chlorophyll and photosynthetic rate and AT7 plants presented the opposite characteristics, AT12 produced more and AT7 produced less seeds than controls. Regarding seed production it was expected to observe better yield when the rosette is larger and the photosynthetic rate is higher. These results could be explained by putative alterations in transport or assimilation of carbohydrates, deserving further investigation.

## Conclusion

The structural differential features exhibited by AtHB7 and AtHB12, together with differential expression patterns resulted in a neo- or sub-functionalization. The analyses discussed above indicated a coordinated regulation of these TFs expression changing at different developmental stages. With the current knowledge it is not clear how this dual coordination is carried out. Transient transformation assays gave further support for these TFs regulating each other. However, the effect of AtHB12 and AtHB7 on each other expression, observed in stable mutants and overexpressors, occurred even when the *35S CaMV* promoter was used. These data strongly indicates that other regulating factors and elements must be involved in such coordinated regulation. Thanks to these loops, AtHB12 and AtHB7 have evolved to finely tune growth and water stress. Further analyses would be necessary to completely understand this interaction remaining in the focus of future research.

## Methods

### Plants material and growth conditions

*Arabidopsis thaliana* ecotype Columbia (Col-0) plants were grown directly on soil in a growth chamber at 22–24°C under long-day photoperiod (16 h light), at an intensity of approximately 150 μE m^-2^ s^-1^, in 8 × 7 cm pots (one plant per pot) during the periods of time indicated in the figures. Plants used for root development analysis were grown in Petri dishes containing Murashige and Skoog basal medium supplemented with vitamins (MS, PhytoTechnologyLaboratories™) and 0.9% (w/v) agar-agar. The dishes were kept at 4°C for 3 days and then transferred to growth chamber conditions and kept for variable periods of time as indicated in the figures legends.

### Genetic constructs and stable transgenic plants generation

Mutant seeds (*athb12*, GK-174E09-013516, presenting a T-DNA insertion at the second exon, and *athb7*, SALK_086222, presenting a T-DNA insertion at the end of the second exon), on Col-0 ecotype background were obtained from the ABRC ( http://www.arabidopsis.org). Homozygous lines were selected after two complete growth cycles.

Double silenced plants (*at12/7*) for *athb12* and *athb7* were generated by transforming plants with an artificial microRNA designed as described by Schwab *et al.*[43]; http://wmd3.weigelworld.org/cgi-bin/webapp.cgi. The PCR *pRS300* vector was used as template and specific oligonucleotides were designed (listed in Additional file 4). The fragment obtained was subcloned in *pGEM T-easy* (Promega) and then cloned in the *Bam*HI site of the *pBI121*. Double *athb7 athb12* silenced plants were named *at12/7.*

*35S::AtHB12* and *35S::AtHB7*constructs were obtained after amplification of both cDNAs using as template total RNA with specific oligonucleotides (Additional file 4) and inserting the amplification products in *Xba*I/*Bam*HI and *Bam*HI/*Sac*I sites of the *pBI121*, respectively. Plants transformed with these constructs were named AT12 and AT7, respectively. Assays were performed at least with three independent lines of each overexpressor genotype.

The promoter constructs in *pKGWFS7* (p*AtHB12:AtHB12::GFP::GUS*, p*AtHB7:AtHB7::GFP::GUS*) were a generous gift of Federico Ariel (ISV- Gif-sur-Yvette- France). Transgenic plants were generated by floral dip transformation of *A. thaliana* wild type plants, Col-0 ecotype, using *Agrobacterium tumefaciens* LBA4404 strain [44].

### Mannitol treatment

Arabidopsis wild type seedlings grown on MS-Agar during 14 days were transferred to liquid MS supplemented with 300 mM mannitol or MS alone as control. After 24 hours the seedlings were harvested for RNA extraction.

### Moderate water stress (MWS) treatment – Water uptake

Plants grown on soil for 21 days were irrigated until saturation. Two days later, the treatment was started: every three days the pots were weighed. The highest weight within all pots was used as reference and water was added to all the pots to reach such reference weight. In this way, all the pots equal their weight every three days. The amount of water added to each pot during the whole treatment was registered to calculate **water uptake** during moderate water stress treatment.

### Water loss analysis

Detached leaves of plants grown in standard conditions were weighed every 20 minutes during 100 min, starting at the detachment moment. The percentage (%) of lost water was expressed as the ratio: [initial weight – weight]/[initial weight] × 100.

### Transient transformation of *Nicotiana benthamiana* leaves

Leaves were transformed by infiltration with a syringe as previously described [35] with cultured *Agrobacterium tumefaciens* LBA4404 transformed with the following constructs: *pBI101.3* as negative control; *pBI121* as positive control; *pAtHB12:AtHB12::GFP::GUS* and p*AtHB7:AtHB7::GFP::GUS* and *35S::AtHB12* and *35S::AtHB7*. Two days after infiltration, samples were frozen and used for RNA extraction.

### RNA extraction and analysis

Total RNA used for qRT-PCR was isolated from Arabidopsis and *N. benthamiana* tissues using the Trizol® reagent (Invitrogen) according to the manufacturer’s instructions. One μg of RNA was reverse-transcribed using oligo(dT)_18_ and M-MLV reverse transcriptase II (Promega). Quantitative real-time PCR (qPCR) was performed with the Mx3000P Multiplex qPCR system (Stratagene, La Jolla, CA) in a 20 μl final volume containing 2 μlSyBr green (4 ×), 8 pmol of each primer, 2 mM MgCl_2_, 10 μl of a 1/15 dilution of the RT reaction and 0.05 μl Platinum Taq (Invitrogen™). Fluorescence was measured at 72°C during 40 cycles. Specific primers were designed (Additional file 4). Quantification of mRNA levels was performed by normalization with the Actins mRNA according to the ΔΔCt method. All the reactions were performed with at least three biological replicates.

### Histochemical GUS staining

*In situ* assays of GUS activity were performed as described before [45]. Whole plants were immersed in a 1 mM 5-bromo-4-chloro-3-indolyl-glucuronic acid solution in 100 mM sodium phosphate pH 7.0 and 0.1% Triton X-100, and, after applying vacuum for 5 min, they were incubated at 37°C for 8 hours. Chlorophyll was cleared from the plant tissues by immersion in 70% ethanol.

### Rosette phenotype analysis

Rosette leaves from different genotypes were detached at the times indicated in the figures and weighed. Leaves were photographed and their area quantified using the ImageJ software [46].

### Stomatal analysis

Arabidopsis leaves of overexpressor and mutant genotypes were used for imprints of the abaxial side. Photographs were taken using optic microscopy (Nikon eclipse E200) with 450× final magnification and a camera Nikon coolpix L810, and then were analysed with the ImageJ software [46].

### Chlorophyll extraction and quantification

Samples of rosette leaves were macerated and 1 ml of 80% acetone was added per 50 mg of tissue. The absorbance of the extracts was measured at 663 and 646 nm with a Nanophotometer (IMPLEN GmbH, Munich, Germany). Total and chlorophyll a, b were calculated according to [47].

### Analysis of CO_2_ exchange and conductance

*A. thaliana* plants from different genotypes were analysed for H_2_O and CO_2_ exchange. Net photosynthetic rates and conductance were measured under saturating light (600 PAR with 10% blue light) using the LI-COR 6400XT Portable Photosynthesis System (Li-Cor Biosciences [ http://www.licor.com/], at optimal CO_2_ concentration (500 μmol mol^-1^).

## Competing interests

The authors declare that they have no competing interests.

## Authors’ contributions

DAR and MC performed all the experimental assays and contributed to the MS writing and Figures design. GB participated in the design and coordination of this work as well as in the MS writing. RLC conceived this study, coordinated the experiments and drafted the MS. All the authors read and approved the MS.

## Supplementary Material

Additional file 1 Water loss of detached leaves during dehydration showed by all the genotypes. Click here for file

Additional file 2 **Protein sequence alignment of AtHB7 and AtHB12.** The positions of the HD, the LZ and the putative sumoylation, phosphorylation, transactivation and unknown motifs are indicated. Click here for file

Additional file 3 **Transcripts levels of *AtHB12 *** **and *AtHB7 *** **in root tissue.** Click here for file

Additional file 4 Oligonucleotides used for cloning and qRT-PCR. Click here for file
