# Supplementary figures and images for: Arabidopsis AtHB7 and AtHB12 evolved divergently to fine tune processes associated with growth and responses to water stress
Source: BMC Plant Biol. 2014 May 31;14:150. doi: 10.1186/1471-2229-14-150 (PMC4064807; doi:10.1186/1471-2229-14-150)

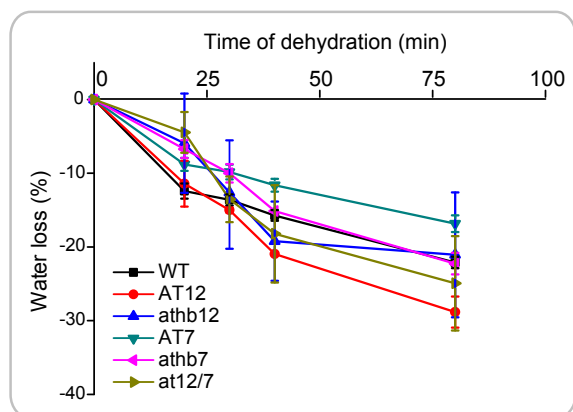

Supplement: Additional file 1 — Water loss of detached leaves during dehydration showed by all the genotypes. [file 1471-2229-14-150-S1.pdf]

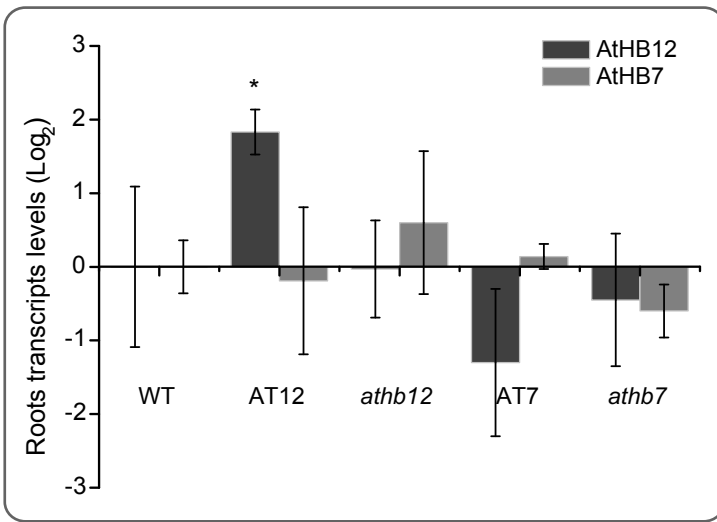

Supplement: Additional file 3 — Transcripts levels of AtHB12 and AtHB7 in root tissue. [file 1471-2229-14-150-S3.pdf]
