# Supplementary material for: Arabidopsis AtHB7 and AtHB12 evolved divergently to fine tune processes associated with growth and responses to water stress
Source: BMC Plant Biol. 2014 May 31;14:150. doi: 10.1186/1471-2229-14-150 (PMC4064807; doi:10.1186/1471-2229-14-150)
Supplement: Additional file 2 — Protein sequence alignment of AtHB7 and AtHB12. The positions of the HD, the LZ and the putative sumoylation, phosphorylation, transactivation and unknown motifs are indicated. [file 1471-2229-14-150-S2.pdf]

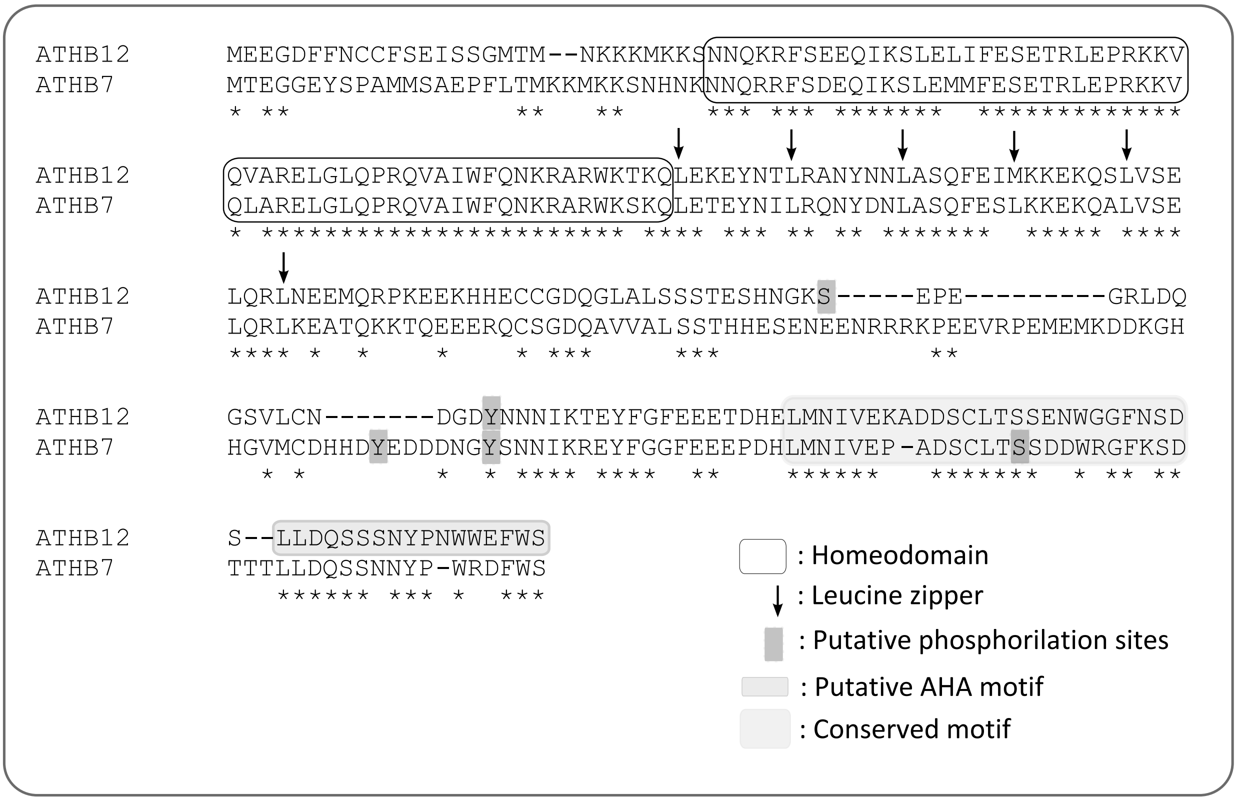
ATHB12 MEEGDFFNCCFSEISSGMTM--NKKKMKKSNNQKRFSEEQIKSLELIFESETRLEPRKKV
ATHB7 MTEGGEYSPAMMSAEPFLTMKKMKKSNHNKNNQRRFSDEQIKSLEMMFESETRLEPRKKV
ATHB12 QVARELGLQPRQVAIWFQNKRARWKTKQLEKEYNTLRANYNNLASQFEIMKKEKQSLVSE
ATHB7 QLARELGLQPRQVAIWFQNKRARWKSKQLETEYNILRQNYDNLASQFESLKKEKQALVSE
ATHB12 LQRLNEEMQRPKEEKHHECCGDQGLALSSSTESHNGKS-----EPE---------GRLDQ
ATHB7 LQRLKEATQKKTQEEERQCSGDQAVVALSSTHHESENEENRRRKPEEVRPEMEMKDDKGH
ATHB12 GSVLCN-------DGDYNNNIKTEYFGFEEETDHELMNIVEKADDSCLTSSENWGGFNSD
ATHB7 HGVMCDHHDYEDDDNGYSNNIKREYFGGFEEEPDHLMNIVEP-ADSCLTSSDDWRGFKSD
ATHB12 S--LLDQSSSNYPNWWEFWS
ATHB7 TTTLLDQSSNNYP-WRDFWS
: Homeodomain
: Leucine zipper
: Putative phosphorilation sites
: Putative AHA motif
: Conserved motif
